# Supplementary material for: Conformational decoupling in acid-sensing ion channels uncovers mechanism and stoichiometry of PcTx1-mediated inhibition
Source: eLife. 2022 Feb 14;11:e73384. doi: 10.7554/eLife.73384 (PMC8871370; doi:10.7554/eLife.73384)
Supplement: Supplementary file 1. — (a). Effect of psalmotoxin-1 (PcTx1) on activation and steady-state desensitization (SSD) of WT and F350L mASIC1a. Data are reported as mean with 95 CI in brackets. (b). SSD and pH-dependent changes in fluorescence for labeled mASIC1a constructs. Data are reported as mean with 95 CI in brackets. (c). Resulting fluorescence change upon application of 300 nM PcTx1 for mASIC1a-labeled constructs. Data are reported as mean with 95 CI in brackets. (d). Change in fluorescence after a 3 min washout of 300 nM PcTx1. Data are reported as mean with 95 CI in brackets. (e). Comparison of current and fluorescence in experiments where 300 nM PcTx1 were applied at pH 7.4. The channels then underwent three 1 min washouts each followed by pH 5.5 stimulus or a single 3 min washout with pH 7.4 followed by a pH 5.5 stimulus. The final pH 5.5 current was normalized to the one at the beginning of the recording, and the fluorescence was analyzed at pH 7.4 right before the final pH 5.5 activation and normalized to deflection induced by PcTx1. Data are reported as mean with 95 CI in brackets. (f) Fluorescence after PcTx1 applications at pH 8.0 followed by different pH regimes (pH 5.5, 7.4, or 8.0) and a washout with pH 7.4. Fluorescence is reported 1 min into the final pH 7.4 application. Data are reported as mean with 95 CI in brackets. (g) Resulting fluorescence change upon peptide application K105C* mASIC1a normalized to the fluorescence response elicited by pH 5.5 application. Big dynorphin (BigDyn) was applied at a concentration of 1 µM, and PcTx1 was applied at a concentration of 300 nM. The fluorescence change was monitored 1 min after application for BigDyn and 30–60 s after application for PcTx1. Data are reported as mean with 95 CI in brackets. (h) Summary of PcTx1 inhibition of mASIC1a constructs. Data are reported as mean with 95 CI in brackets. (i) pH sensitivity of activation and SSD for mASIC1a constructs. Data are reported as mean with 95 CI in brackets. [file elife-73384-supp1.docx]

**Supplementary Files for:**

**Conformational decoupling in acid-sensing ion channels uncovers mechanism and stoichiometry of PcTx1-mediated inhibition**

Stephanie A. Heusser^#^, Christian B. Borg^#^, Janne M. Colding, Stephan A. Pless

^#^ Contributed equally

**Contents:**

Supplementary Files 1a-i

**Supplementary File 1a.**

|  | **WT** | | | **F350L** | | |
| --- | --- | --- | --- | --- | --- | --- |
| **PcTx1 (nM)** | **pH_50_** | **Hill** | **n** | **pH_50_** | **Hill** | **n** |
| **Activation** | | | | | | |
| 0 | 6.68 (6.64, 6.72) | 6.16 (2.74, 9.58) | 18 | 6.26 (6.17, 6.35) | 3.61 (2.75, 4.47) | 12 |
| 30 | 7.21 (7.12, 7.30) | 2.72 (2.08, 3.36) | 7 | 6.20 (6.06, 6.36) | 3.76 (3.02, 4.53) | 7 |
| **Steady-state desensitization** | | | | | | |
| 0 | 7.28 (7.22, 7.33) | 14.31 (6.20, 22.43) | 6 | 7.12 (7.09, 7.16) | 19.51 (12.79, 26.22) | 5 |
| 30 | 7.68 (7.63, 7.72) | 17.15 (7.04, 27.27) | 9 | 7.28 (7.21, 7.36) | 18.78 (5.36, 32.20) | 7 |

**Supplementary File 1b.**

|  | **pH_50_** | **Hill** | **n** |
| --- | --- | --- | --- |
| **Steady-state desensitization** | | | |
| K105C* | 7.06 (7.02, 7.09) | 13.96 (10.88, 17.03) | 4 |
| WT*/WT*/WT* | 7.14 (7.12, 7.16) | 11.56 (7.87, 15.25) | 6 |
| WT*/F350L*/WT* | 7.13 (7.07, 7.19) | 7.84 (4.73, 10.95) | 4 |
| WT*/F350L*/F350L* | 7.06 (7.02, 7.10) | 15.04 (6.90, 23.18) | 4 |
| K105C*F350L | 6.99 (6.96, 7.03) | 11.66 (7.34, 15.98) | 4 |
| V80C* | 7.17 (7.13, 7.21) | 11.95 (5.34, 18.57) | 4 |
| V80C*F350L | 7.31 (6.72, 7.89) | 8.56 (0.03, 17.15) | 4 |
| **Fluorescence signal** | | | |
| K105C* | 7.11 (7.15, 7.06) | 7.24 (5.40, 9.07) | 5 |
| WT*/WT*/WT* | 7.15 (7.18, 7.12) | 8.07 (5.54, 10.59) | 6 |
| WT*/F350L*/WT* | 7.14 (7.11, 7.16) | 9.09 (5.06, 13.12) | 5 |
| WT*/F350L*/F350L* | 7.07 (7.15, 7.00) | 7.72 (3.70, 11.74) | 4 |
| K105C*F350L | 6.95 (6.86, 7.52) | 6.69 (3.91, 9.47) | 6 |
| V80C* | 7.36 (7.30, 7.42) | 9.41 (6.62, 12.21) | 4 |
| V80C*F350L | 7.16 (7.14, 7.18) | 13.87 (8.68, 19.06) | 5 |

**Supplementary File 1c.**

| **Construct** | **Application pH** | **ΔF_PcTx1_/ΔF_pH_ _5.5_** | **n** |
| --- | --- | --- | --- |
| K105C* | 7.4 | 1.13 (1.09, 1.18) | 14 |
| WT*/WT*/WT* | 7.4 | 1.25 (1.01, 1.50) | 6 |
| WT*/F350L*/WT* | 7.4 | 0.99 (0.86, 1.12) | 8 |
| WT*/F350L*/F350L* | 7.4 | 0.70 (0.46, 0.95) | 5 |
| K105C*/F350L | 7.4 | 0.019 (0.001, 0,04) | 6 |
| K105C*/F350L | 7.3 | 0.464 (0.24, 0.68) | 5 |
| V80C* | 7.7 | -0.86 (-0.90, -0.81) | 13 |
| V80C*/F350L | 7.4 | -0.70 (-0.87, -0.53) | 8 |

**Supplementary File 1d.**

| **Construct** | **Wash pH (3 min)** | **ΔF_3min_/ΔF _PcTx1_** | **n** |
| --- | --- | --- | --- |
| K105C* | 7.4 | 0.68 (0.37, 0.98) | 6 |
|  | 8.4 | 0.28 (0.19, 0.37) | 4 |
|  | mix | 0.25 (0.08, 0.42) | 4 |
| WT*/WT*/WT* | 7.4 | 0.83 (0.78, 0.88) | 6 |
| WT*/F350L*/WT* | 7.4 | 0.49 (0.39, 0.60) | 8 |
| WT*/F350L*/F350L* | 7.4 | 0.15 (0.33, 0.04) | 5 |
| K105C*/F350L | 7.4 | 0.03 (-0.01, 0.08 ) | 5 |
| V80C* | 7.7 | -0.70 (-0.96, -0.44) | 5 |
|  | 8.4 | -0.41 (-0.60, -0.23) | 4 |
|  | mix | -0.56 (-0.79, -0.33) | 4 |
| V80C*/F350L | 7.4 | -0.059 (-0.13, 0.01) | 6 |

**Supplementary File 1e.**

| **Treatment** | **I_7.4_/I_PcTx1_** | **ΔF_7.4_/ ΔF _PcTx1_** | **n** |
| --- | --- | --- | --- |
| 3 min washout | 0.79 (0.69, 0.89) | 0.73 (0.49, 0.96) | 6 |
| Multiple activations | 0.83 (0.78, 0.88) | 0.68 (0.54, 0.82) | 4 |

**Supplementary File 1f.**

| **Treatment** | **ΔF_7.4_/ΔF_5.5_** | **n** |
| --- | --- | --- |
| PcTx1 at pH 8.0 | -0.08 (-0,14, -0.02) | 7 |
| 7.4 after 5.5 | 0.95 (0.81, 1.08) | 6 |
| 7.4 after 7.4 | 0.69 (0.60, 0.78) | 4 |
| 7.4 after 8.0 | 0.11 (0.01, 0.23) | 4 |

**Supplementary File 1g.**

| **Treatment** | **ΔF_peptide_/ΔF_pH_ _5.5_** | **n** |
| --- | --- | --- |
| BigDyn (Control) | -0.84 (-1.05, -0.64) | 6 |
| BigDyn post PcTx1 | 0.50 (0.18, 0.83) | 6 |
| PcTx1 (Control) | 0.86 (0.67, 1.06) | 6 |
| PcTx1 post BigDyn | 0.77 (0.61, 0.93) | 4 |
| PcTx1 (Control) | 1.20 (1.05, 1.34) | 6 |
| PcTx1 post PcTx1 | 1.17 (1.06, 1.28) | 6 |

**Supplementary File 1h.**

|  | **IC_50_** | **Hill** |  |
| --- | --- | --- | --- |
| **Construct** | **Mean (nM)** | **Mean** | **n** |
| WT | 0.6 (0.4, 0.9) | 0.9 (0.7, 1.2) | 9-14 |
| F350L | 977.2 (-, -) | 11.2 (- , -) | 4-7 |
| WT/WT/WT | 6.5 (5.2, 9.0) | 1.1 (0.9, 1.3) | 6-9 |
| F350L/WT/WT | 7.3 (5.9, 9.0) | 1.1 (0.9, 1.4) | 5-6 |
| WT/F350L/WT | 6.6 (5.7, 7.7) | 1.3 (1.0, 1.5) | 5-7 |
| WT/WT/F350L | 4.2 (3.2, 4.8) | 1.0 (0.9, 1.2) | 5-7 |
| F350L/F350L/WT | 94.6 (74.4, 120.1) | 0.9 (0.7, 1.1) | 4-6 |
| F350L/WT/F350L | 289.9 (218.1, 387.2) | 0.8 (0.7, 1.1) | 6-11 |
| WT/F350L/F350L | 108.0 (834, 141.8) | 0.8 (0.7, 1.0) | 4-7 |
| F350L/F350L/F350L | 797.1 (654.1, 962.4) | 2.6 (1.7, -) | 4-6 |

**Supplementary File 1i.**

| **Construct** | **pH_50_** | **Hill** | **n** |
| --- | --- | --- | --- |
| **Activation** | | | |
| WT | 6.69 (6.65, 6.73) | 7.70 (5.88, 9.53) | 10 |
| WT/WT/WT | 6.72 (6.69, 6.75) | 6.31 (4.92, 7.71) | 10 |
| F350L/WT/WT | 6.59 (6.53, 6.64) | 5.80 (2.87, 8.74) | 8 |
| WT/F350L/WT | 6.62 (6.58, 6.65) | 8.82 (6.40, 11.24) | 13 |
| WT/WT/F350L | 6.62 (6.58, 6.66) | 7.72 (4.79, 10.66) | 10 |
| F350L/F350L/WT | 6.47 (6.44, 6.50) | 6.29 (4.26, 8.32) | 13 |
| F350L/WT/F350L | 6.43 (6.39, 6.46) | 5.34 (4.75, 5.92) | 8 |
| WT/F350L/F350L | 6.44 (6.37, 6.50) | 6.96 (4.15, 9.77) | 10 |
| F350L/F350L/F350L | 6.18 (6.11, 6.26) | 3.86 (2.56, 5.17) | 7 |
| F350L | 6.31 (6.18, 6.43) | 4.09 (2.87, 5.30) | 7 |
| **Steady-state desensitisation** | | | |
| WT | 7.27 (7.25, 7.30) | 14.15 (10.20,18.09) | 11 |
| WT/WT/WT | 7.24 (7.22, 7.25) | 12.11 (10.72, 13.50) | 5 |
| WT/F350L/WT | 7.24 (7.19, 7.29) | 13.79 (8.62, 18.97) | 5 |
| WT/F350L/F350L | 7.17 (7.14, 7.20) | 9.09 (5.77, 12.42) | 6 |
| F350L/F350L/F350L | 7.16 (7.11, 7.21) | 12.45 (4.60, 20.29) | 4 |
| F350L | 7.11 (7.09, 7.14) | 12.36 (8.96, 15.76) | 7 |
